# Mulinane and Azorellane Diterpenoid Biomarkers by GC-MS from a Representative Apiaceae (Umbelliferae) Species of the Andes

**DOI:** 10.3390/molecules24040684

**Published:** 2019-02-14

**Authors:** Bernd R.T. Simoneit, Daniel R. Oros, Rudolf Jaffé, Alexandra Didyk-Peña, Carlos Areche, Beatriz Sepúlveda, Borys M. Didyk

**Affiliations:** 1 Department of Chemistry, College of Science, Oregon State University, Corvallis, OR 97331, USA; 2 Consultant, 72 Marina Lakes Drive, Richmond, CA 94804, USA; daniel.r.oros@gmail.com; 3 Southeast Environmental Research Center and Department of Chemistry and Biochemistry, Florida International University, 3000 NE 151st Street, North Miami, FL 33181, USA; jaffer@fiu.edu; 4 Escuela de Química y Farmacia, Facultad de Medicina, Universidad Andres Bello, Viña del Mar 2520000, Chile; 5 Departamento de Química, Facultad de Ciencias, Universidad de Chile, Casilla 653, Santiago 8320000, Chile; areche@uchile.cl; 6 Departamento de Ciencias Químicas, Universidad Andres Bello, Viña del Mar 2520000, Chile; bsepulveda@uc.cl; 7 Consultant, Casilla 942, Viña del Mar 2520000, Chile; bmdidyk@gmail.com

**Keywords:** *Azorella compacta*, diterpenoids, GC-MS, standards

## Abstract

Extracts of bled resin from *Azorella compacta*, of the Azorelloideae family from the Andes (>4000 m), were analyzed by gas chromatography-mass spectrometry. The mass spectra of the dominant compounds of the resin and its hydrogenation products were documented. The most abundant compounds were oxygenated diterpenoids, namely mulinadien-20-oic (Δ^11,13^ and Δ^11,14^) acids, azorell-13-en-20-oic acid, 13α,14β-dihydroxymulin-11-en-20-oic acid, and azorellanol, with a group of azorellenes and mulinadienes. The mass spectra of the novel diterpenoid hydrocarbons with the azorellane and mulinane skeletons were also presented. This study documents the molecular diversity of these diterpenoid classes, and could be of great utility for future organic geochemical, environmental, archeological, pharmaceutical, and forensic chemistry studies.

## 1. Introduction

Natural products or their derivatives in the ambient environment or geological record are used by organic geochemists as tracers for sources, transport and alteration processes of organic matter, e.g., [1,2,3,4,5,6,7]. Remote locales, for example, the high Andes region in South America, are ideal sites as pristine references for background studies of such processes. An early report on the lipids and biomarkers in sediment from Lake Lejia in a caldera in Antofagasta, Chile, found traces of diterpenoids typical of resinous vegetation [8]. The region is barren of trees, especially gymnosperms that produce resin, and potential sources such as the Araucariaceae grow far to the south [9]. Subsequently, we reported on the lipid and resin composition of a dried sample of *Laretia compacta* (common name Llareta) from San Pedro de Atacama [10]. The dried material had a strong resinous odor and thus was implied as a possible source of diterpenoids in the Altiplano environment. The extract yield of the whole plant based on dry weight comprised 4.2 mg/g (0.2%) lipids and 2.5 mg/g (0.12%) terpenoids [10]. The terpenoid distribution consisted of mono-, sesqui- and diterpenoids, and the latter are of interest here because tri- and tetracyclic diterpenoids were the dominant compounds.

*Laretia compacta* is now called *Azorella compacta* of the Apiaceae:Azorelloideae family, also known as Umbelliferae [11]. These plants grow slowly at high altitudes (>4000 m) in the northern Andes of Chile and Peru as dense pillow-like structures close to the ground with subterranean stems and roots. The Azorelloideae family evolved from the first Umbelliferae during the Cretaceous, e.g., [12,13]. 

**Figure 1:**
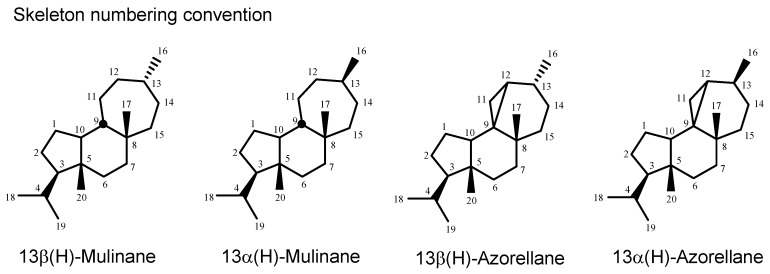
Carbon numbering convention for the mulinane and azorellane skeletons.

*Azorella* sp. have historically been used on the Altiplano as firewood and cooking fuel by local cultures, and later in early industrial and mining activities as fuel, which contributed to its scarcity and current protection. The species is now more common, and represents the dominant biomass in these Andean environments. The plants and resin exudates have been used by Andean cultures for medicinal purposes where, for example, hot water infusions serve as herbal remedies for various ailments [14,15]. Consequently, natural product characterization and pharmacological studies have been carried out on *A. compacta* and related species. Numerous investigations reported structure determinations and pharmacological potential studies of the resin compounds, that were isolated and purified from the major Andean Apiaceae species, namely *Mulinum* and *Azorella* [16,17,18,19,20,21,22,23,24,25,26,27,28,29,30,31,32,33,34,35]. This has led to a description of mainly two novel diterpenoid skeletons among the natural products, specifically the mulinanes and azorellanes (Figure 1), with the assignment of their skeletal numbering conventions [16,21]. The biosynthetic pathway for the mulinane and azorellane diterpenoids has been proposed as starting from a phytatetraene [24]. Laboratory syntheses of polar mulinane diterpenoids have also recently been reported [36,37].

Here, we characterize the molecular composition of *Azorella compacta* resin based on mass spectrometric interpretation, and correlation and comparison with known standards. We report the mass spectra and gas chromatography-mass spectrometry (GC-MS) characteristics of the dominant compounds of the resin and of the hydrogenated resin as the free and derivatized products. This information is unique, because prior reports in the natural product literature only provided mass spectrometric data on some compounds. Our data further documents the molecular diversity of this novel diterpenoid class (mulinane and azorellane), information which could be of great utility for future organic geochemical, environmental, archeological, pharmaceutical, and forensic chemistry studies. 

## 2. Results and Discussion

The relative composition of the fresh resin was 1% sesquiterpenoid and 99% diterpenoids, excluding a low amount of bornyl acetate (<0.5%). No lipid components from plant wax were detected. It is of interest to note the breakdown of the diterpenoids into 19% hydrocarbons and 80% polar oxygenated compounds, and the same proportions were retained upon hydrogenation. This observation was unexpected because most bled conifer resins are characterized by higher oxygenated diterpenoid contents (Simoneit, unpublished data). 

**Figure 2:**
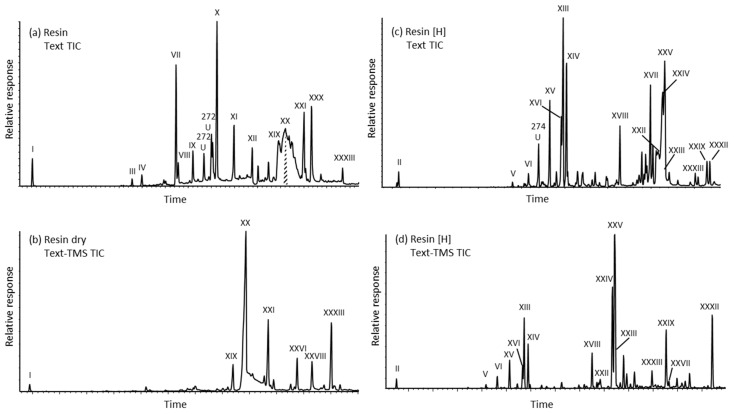
Examples of GC-MS total ion current traces for *A. compacta* resin and hydrogenated resin analyzed as: (**a**,**c**) total extracts, and (**b**,**d**) silylated extracts. Roman numerals refer to structures in Table 1 and Appendix A.

Typical examples of GC-MS total ion current traces for *A. compacta* resin and hydrogenated resin indicated the molecular complexity of the compound mixtures (Figure 2). The poor resolution of the underivatized resin acids (cf. Figure 2a) was due to their polarity. Sample components were classified into three groups of compounds, sesquiterpenoid, diterpenoid hydrocarbons and oxygenated diterpenoids, and are discussed as such. Only one sesquiterpenoid, namely spathulenol (I, all structures are shown in Appendix A), which hydrogenates to two isomers of spathulanol (II), was observed. The relative concentrations of all identified compounds are listed in Table 1. 

### 2.1. Diterpenoid Hydrocarbons

**Figure 3:**
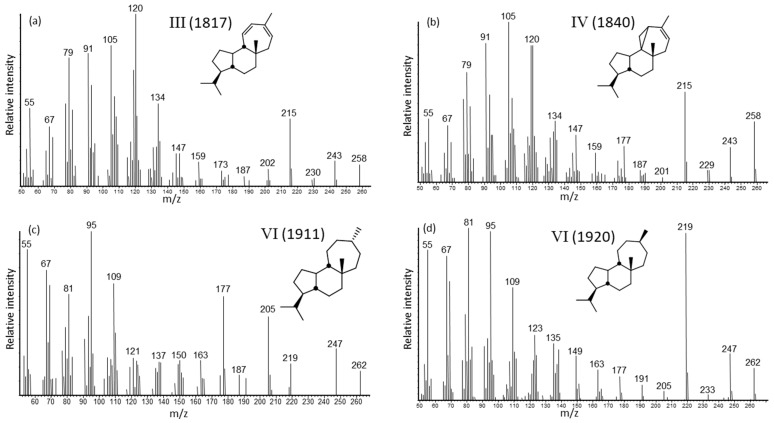

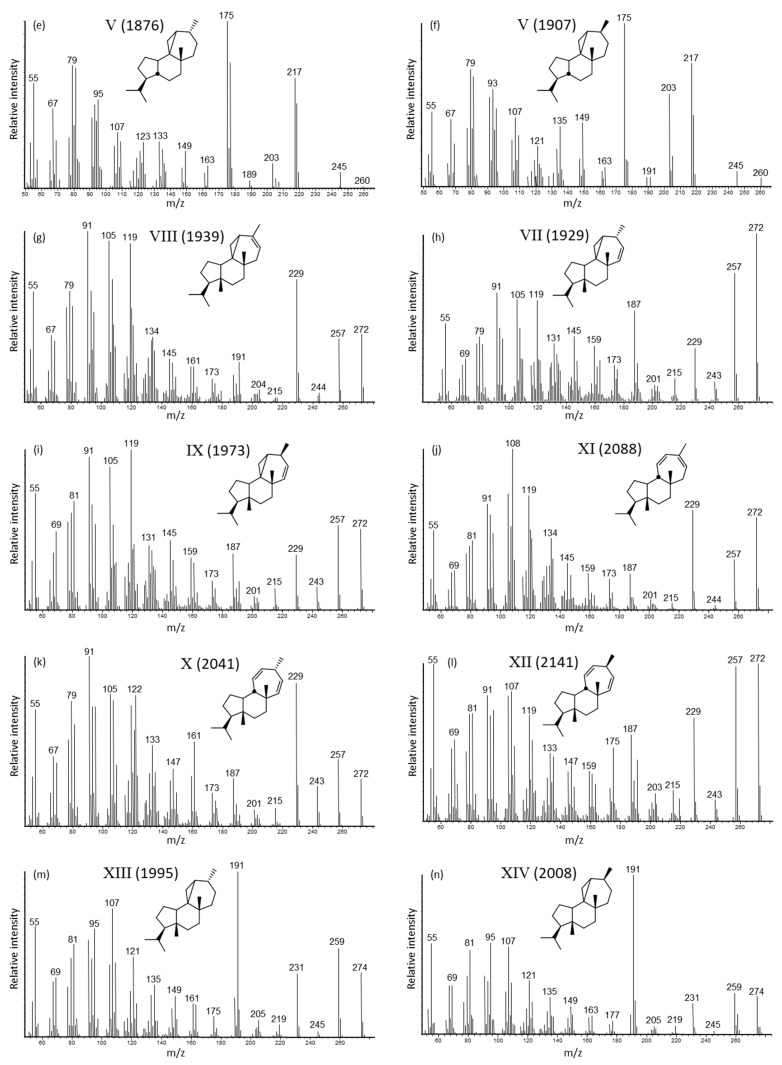

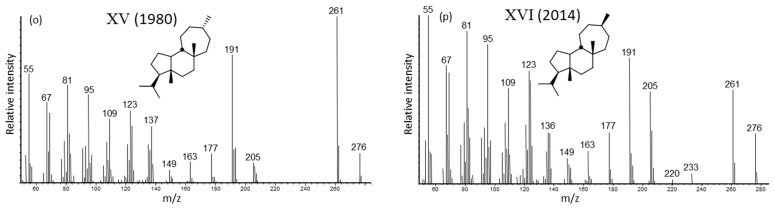
Mass spectra of the diterpenoid hydrocarbons in *A. compacta* resins. The KI values relative to *n*-alkanes on a DB-5 column are shown in parentheses on each mass spectrum: (**a**) 20-normulina-11,13-diene (III), (**b**) 20-norazorell-13-ene (IV), (**c**) 13β(H)-20-normulinane (VI), (**d**) 13α(H)-20-normulinane (VI), (**e**) 13β(H)-20-norazorellane (V), (**f**) 13α(H)-20-norazorellane (V), (**g**) azorell-13-ene (VIII), (**h**) 13β(H)-azorell-14-ene (VII), (**i**) 13α(H)-azorell-14-ene (IX), (**j**) mulina-11,13-diene (XI), (**k**) 13β(H)-mulina-11,14-diene (X), (**l**) 13α(H)-mulina-11,14-diene (XII), (**m**) 13β(H)-azorellane (XIII), (**n**) 13α(H)-azorellane (XIV), (**o**) 13β(H)-mulinane (XV), and (**p**) 13α(H)-mulinane (XVI).

There were two norditerpenes, 20-normulina-11,13-diene (III) and 20-norazorell-13-ene (IV), which upon hydrogenation yielded four isomers, namely 20-normulinane (VI) and 20-norazorellane (V) both as the 13β(H) and 13α(H)-epimers (Figure 3a–f, also see Figure SM-2b). We inferred the absence of C-20 for these novel norditerpenes, rather than C-16 or C-17, based on the facile elimination of the functionalized C-20 group in the mass spectra of the acid standards discussed below. The configuration at C-5 is most likely 5β(H), but based on the preferential loss or absence of a C-20 functional group, could also be represented as 5α(H). 

There were six isomers of C_20_ diterpene hydrocarbons, which after hydrogenation yielded four isomers of the saturated diterpanes. The interpretations of the mass spectra as mulina-11,13-diene (XI), 13β(H)-mulina-11,14-diene (X) and 13α(H)-mulina-11,14-diene (XII) (Figure 3j–l) were inferred from the literature [32], although the match was poor. The identifications of azorell-13-ene (VIII), 13β(H)-azorell-14-ene (VII) and 13α(H)-azorell-14-ene (IX) were based on the GC elution order and interpretations of the mass spectrometric fragmentation patterns (Figure 3g–i; details are given in Figure SM-2b) when compared to the C-20 functionalized standards.

Upon hydrogenation to the mulinane and azorellane skeletons (Figure 1), we obtained two isomers, i.e., 13α(H) and 13β(H), for the position of the methyl group at C-13. We assigned the elution order as was observed for the α and β kaurane and phyllocladane standards [38,39], that is the 13β(H) isomer eluted prior to the 13α(H) epimer. The diterpanes, namely 13β(H)-azorellane (XIII), 13α(H)-azorellane (XIV), 13β(H)-mulinane (XV), and 13α(H)-mulinane (XVI), have similar mass spectra (Figure 3m–p), but different molecular weights. The key ion for all is *m*/*z* 191, with M-C_3_H_7_ (*m*/*z* 231) for the azorellanes and M-C_5_H_11_ (*m*/*z* 205) for the mulinanes (cf. Figure SM-2b). These four compounds may be potential biomarkers for the geologic record and of interest to organic geochemistry. The mass spectra of both C_20_ hydrocarbon groups are the first report of these novel parent diterpenoid skeletons, and they do not match with any mass spectra of other reported tri- and tetracyclic diterpanes (e.g., abietanes, atisanes, pimaranes, kauranes, phyllocladanes, etc.).

### 2.2. Oxygenated Diterpenoids

**Figure 4:**
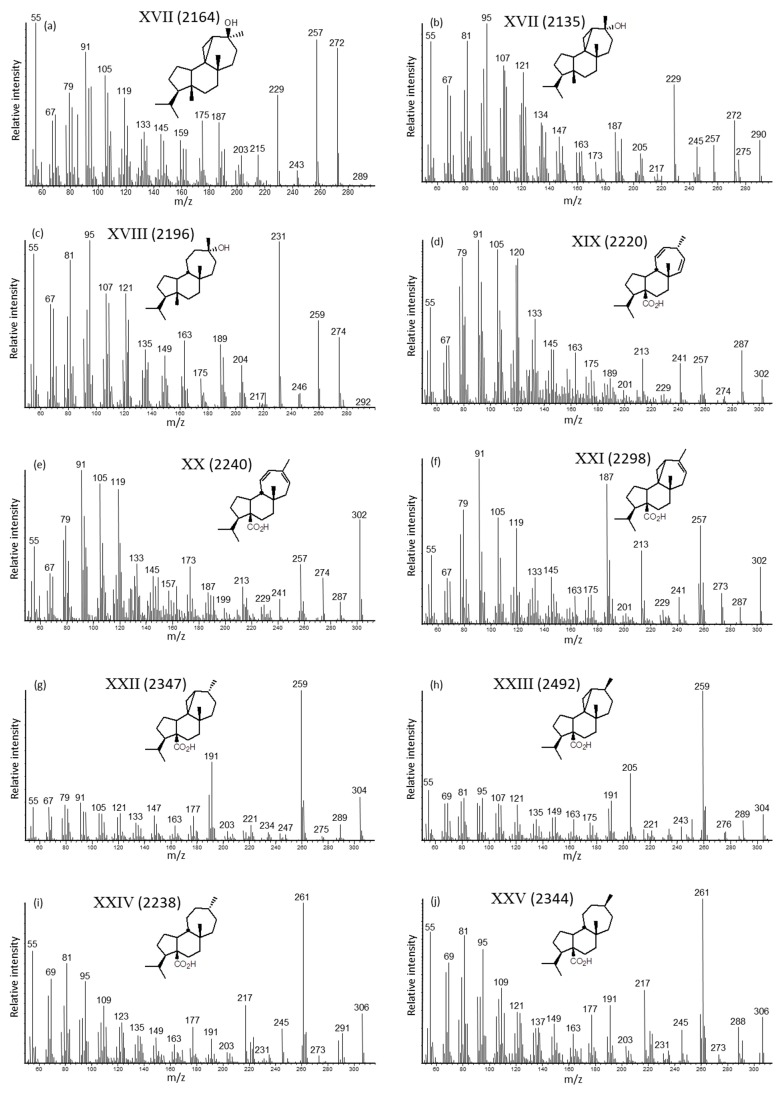

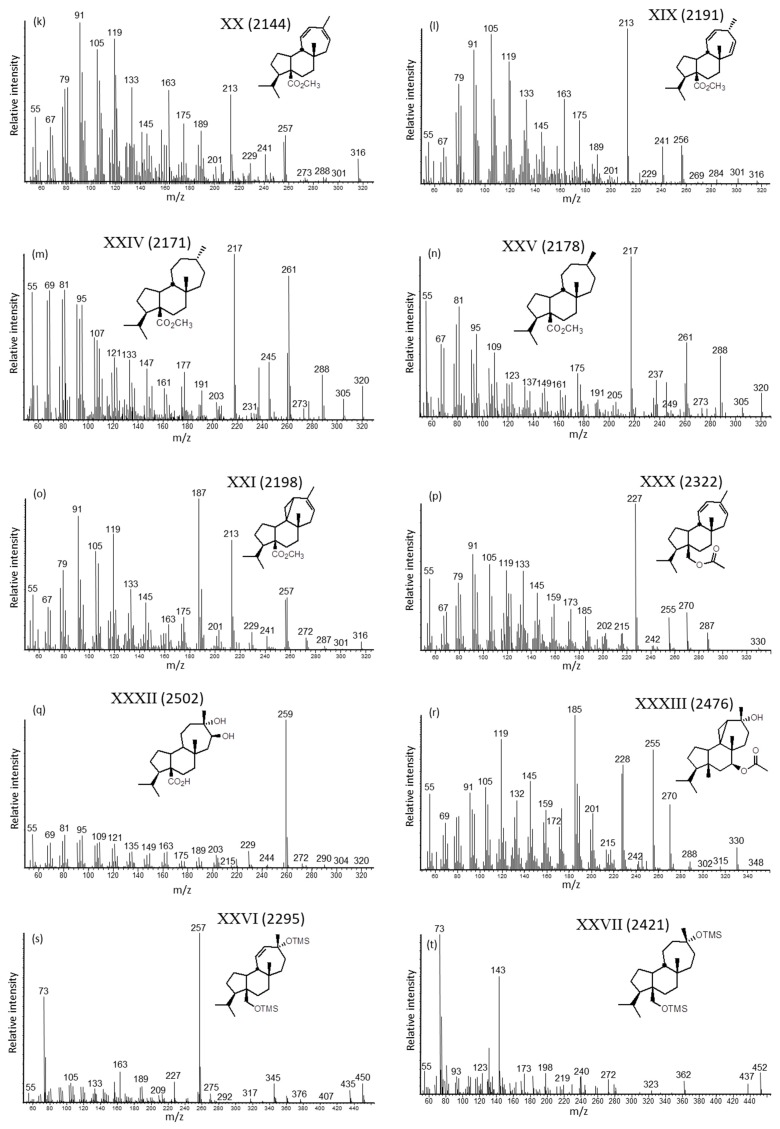

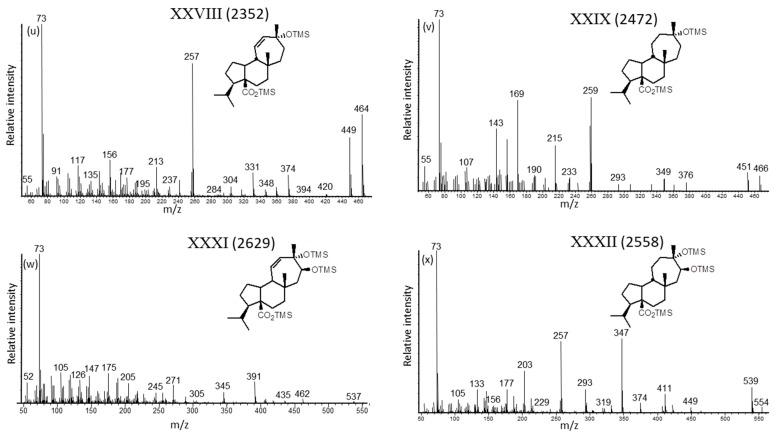
Mass spectra of the oxygenated diterpenoids in *A. compacta* resins, including KI values shown in parentheses: (**a**) 13β-hydroxyazorellane (XVII), (**b**) 13α-hydroxyazorellane (XVII), (**c**) 13α-hydroxymulinane (XVIII), (**d**) 13β(H)-mulina-11,14-dien-20-oic acid (XIX, standard), (**e**) mulina-11,13-dien-20-oic acid (XX, standard), (**f**) azorell-13-en-20-oic acid (XXI, standard), (**g**) 13β(H)-azorellan-20-oic acid (XXII, standard), (**h**) 13α(H)-azorellan-20-oic acid (XXIII, standard), (**i**) 13β(H)-mulinan-20-oic acid (XXIV, standard), (**j**) 13α(H)-mulinan-20-oic acid (XXV, standard), (**k**) methyl mulina-11,13-dien-20-oate (XX, standard), (**l**) methyl 13β(H)-mulina-11,14-dien-20-oate (XIX, standard), (**m**) methyl 13β(H)-mulinan-20-oate (XXIV, standard), (**n**) methyl 13α(H)-mulinan-20-oate (XXV, standard), (**o**) methyl azorell-13-en-20-oate (XXI, standard), (**p**) 20-acetoxymulina-11,13-diene (XXX), (**q**) 13α,14β-dihydroxymulinan-20-oic acid (XXXII), (**r**) azorellanol (XXXIII, standard), (**s**) mulinol-diTMS (XXVI), (**t**) dihydomulinol-diTMS (XXVII), (**u**) 13α-hydroxymulin-11-en-20-oic acid-diTMS (XXVIII, mulinolic acid-diTMS, standard), (**v**) 13α-hydroxymulinan-20-oic acid-diTMS (XXIX, standard), (**w**) 13α,14β-dihydroxymulin-11-en-20-oic acid-triTMS (XXXI), and (**x**) 13α,14β-dihydroxymulinan-20-oic acid-triTMS (XXXII).

The oxygenated diterpenoids comprised mainly mulina-11,13-dien-20-oic acid (XX) with the isomers 13β(H)-mulina-11,14-dien-20-oic (XIX) and azorell-13-en-20-oic (XXI) acids. Their identification was based on comparison of the mass spectra of standard natural products as the free and derivatized compounds (Figure 4d–f,k–o and Figure SM-1o–p, respectively), and their respective GC retention indices. One characteristic of the mass spectra of all C-20 carboxylic acids and C-20 methyl or TMS carboxylates is the facile loss of that moiety from the respective molecular ion (cf. Figure SM-2a). 

Significant amounts of 20-acetoxymulina-11,13-diene (XXX), azorellanol (7β-acetoxy-13α-hydroxyazorellane, XXXIII), and 13α,14β-dihydroxymulin-11-en-20-oic acid (XXXI) were also identified. The major fragment ions of the mass spectrum of 20-acetoxymulina-11,13-diene (Figure 4p) matched with those listed in the literature for its direct insertion probe mass spectrum [26]. The mass spectrum of the azorellanol standard (Figure 4r and Figure SM-1v as the TMS derivative) matched with its literature listing [21]. There was also a trace of the tentatively identified 13-*epi*-azorellanol (Figure SM-1k), which eluted prior to azorellanol. The structure of 13α,14β-dihydroxymulin-11-en-20-oic acid (Figure 4w) was based on correlation with the standard 13α-hydroxy-14-oxomulin-11-en-20-oic acid (XXXIV, Figure SM-1j,w,z). Mulinol (13α,20-dihydroxymulin-11-ene, XXVI) and 13α-hydroxymulin-11-en-20-oic acid (mulinolic acid, XXVIII) were observed as minor components. The mass spectrum of mulinol (Figure 4s) was correlated with that of its hydrogenation product (Figure 4v) and the published MS listing [20]. The mass spectra of mulinolic acid matched with those of the standard (Figure 4u and Figure SM-1d,h).

The hydrogenated resin sample contained the corresponding saturated diterpenoids (Figure 2c,d). As discussed above for the hydrocarbons, the elution order assumed for the dominant 13β(H)- and 13α(H)-mulinan-20-oic acids (XXIV and XXV, respectively) was beta before alpha (Figure 4i,j,m,n and Figure SM-1r,s, respectively). The same was the case for the 13β(H)- and 13α(H)-azorellan-20-oic acids (XXII and XXIII, respectively) and their mass spectra are shown in Figure 4g,h and Figure SM-1r,s, respectively (refer to Figure SM-2a). These diterpanoid acids may be potential diagenetic products in the geologic record and thus are of interest to organic geochemistry. The hydrogenation products of mulinol and dihydroxymulin-11-en-20-oic acid were also detectable as dihydromulinol (XXVII, Figure 4t) and 13α,14β-dihydroxymulinan-20-oic acid (XXXII, Figure 4x). 

## 3. Samples and Experimental Methods

A sample of *A. compacta* resin was obtained by cutting to bleed an *Azorella compacta* plant growing off the road B245 from El Tatio to San Pedro de Atacama in the Paso la Vizcacha (Chile, Region II, 22°22′34″S, 68°1′0″W, altitude 4556 m). 

The fresh resin was split into two parts, one dissolved completely in dichloromethane/methanol (DCM/MeOH, 3:1 *v*/*v*), and the duplicate sample was dissolved in *n*-hexane. Aliquots (50 µL) of these total extracts were converted to trimethylsilyl derivatives by reaction with N,O-bis-(trimethylsilyl)trifluoroacetamide (BSTFA, Sigma-Aldrich, St. Louis, MO, USA) and pyridine for 3 h at 70 °C, e.g., [40]. Prior to analysis, the excess silylating reagent was evaporated by nitrogen blow down and the sample mixture dissolved in an equivalent volume of *n*-hexane. Other aliquots of sample solutions were treated with trimethylsilyldiazomethane (2M in hexane, Sigma-Aldrich) at room temperature for 30 min to methylate carboxylic acids [41]. The excess reagent was reacted with concentrated acetic acid, the solution dried by nitrogen blow down, and the products dissolved in DCM/MeOH (3:1) for analysis. 

The following reference standards were also derivatized and analyzed by these methods: spathulenol (I), mulina-11,13-dien-20-oic acid (XX), azorell-13-en-20-oic acid (XXI), 13α-hydroxymulin-11-en-20-oic acid (XXVIII), azorellanol (XXXIII), 13α-hydroxy-14-oxomulin-11-en-20-oic acid (XXXIV), 7β,13α-dihydroxymulin-11-ene (XXXV), 15α-acetoxymulina-11,13-dien-20-oic acid (XXXVI), and 18-acetoxymulina-11,13-diene-16,20-dioic acid (XXXVII).

Aliquots of the total extracts and mulina-11,13-dien-20-oic acid were hydrogenated. The samples were diluted with DCM/MeOH (3:1) in a round bottom flask (50 mL) with a magnetic stir bar and PtO_2_ (Arcos Organics, Fisher, Pittsburgh, PA, USA, 83% Pt) as catalyst. H_2_ at atmospheric pressure was installed with a latex balloon (500 mL) over the neck of the flask and hydrogenation allowed to proceed at room temperature for 8 h. The catalyst was filtered off and the sample concentrated by nitrogen blow down for analysis.

The gas chromatography-mass spectrometry (GC-MS) analyses of the total extracts and aliquots of the derivatized and hydrogenated total extracts (typical injection volume 1 µL) were performed on a Hewlett-Packard model 6890 GC coupled to a Hewlett-Packard model 5973 MSD (Palo Alto, CA, USA)). Separation of compounds was achieved on a fused silica capillary column coated with DB-5MS (Agilent, Santa Clara, CA, USA) 30 m × 0.25 mm i.d., 0.25 µm film thickness). The GC operating conditions were as follows: temperature held at 65 °C for 2 min, increased from 65 to 300 °C at a rate of 6 °C min^−1^, and final isothermal held at 300 °C for 20 min. Helium was used as carrier gas. The samples were injected splitless with the injector temperature at 280 °C. The mass spectrometer was operated in the electron impact mode at 70 eV and scanned from 50 to 650 da. Data were acquired and processed with the Chemstation software (Hewlett-Packard, Palo Alto, CA, USA). Compounds were identified by comparison of mass spectra and retention times with those of authentic standards, with literature data, and interpretation of mass spectrometric fragmentation patterns of unknowns. The Kovats [42] GC retention indices (KI) relative to *n*-alkanes are shown on each mass spectrum and are determined for elution on a DB-5 capillary column.

The extract of the dried plant (prior report by [10]) was reanalyzed. The dominant diterpenoids were the same as found in the new resin sample and are not discussed further.

## 4. Conclusions

In this study, we document the molecular characteristics and diversity of novel diterpenoid classes of natural products, namely mulinanes and azorellanes, from resin of the Andean Apiaceae plant species *Azorella compacta*. The dominant compounds were oxygenated diterpenoids, mainly mulinadien-20-oic (Δ^11,13^ and Δ^11,14^) acids, azorell-13-en-20-oic acid, 13α,14β-dihydroxymulin-11-en-20-oic acid, and azorellanol, as confirmed by standards. The diterpenoid hydrocarbons included mulinadienes and azorellenes, which are novel compounds of potential interest to organic geochemistry. The mulinane and azorellane diterpenoid hydrocarbons, resulting from the hydrogenation of the resin, may be potential biomarkers for the geologic record, while the corresponding oxygenated diterpenoids may be applied for environmental studies. 

## Figures and Tables

**Table 1:** Relative concentrations of the major compounds identified in resin and hydrogenated resin of *Azorella compacta*.

Structure Number ^a^	Compound	Composition ^b^	MW	ID ^c^	Resin (n = 7)	Hydrogenated Resin (n = 4)
I	Spathulenol	C_15_H_24_O	220	S	1.4	
II	Spathulanol	C_15_H_26_O	222	S		3
III	20-Normulina-11,13-diene	C_19_H_30_	258	T	0.5	
IV	20-Norazorell-13-ene	C_19_H_30_	258	T	1	
V	20-Norazorellane, α + β	C_19_H_32_	260	T		1
VI	20-Normulinane, α + β	C_19_H_34_	262	T		0.3
VII	13β(H)-Azorell-14-ene	C_20_H_32_	272	T	9	
VIII	Azorell-13-ene	C_20_H_32_	272	I	2	
IX	13α(H)-Azorell-14-ene	C_20_H_32_	272	T	2.5	
X	13β(H)-Mulina-11,14-diene	C_20_H_32_	272	I	16	
XI	Mulina-11,13-diene	C_20_H_32_	272	L	7	
XII	13α(H)-Mulina-11,14-diene	C_20_H_32_	272	I	4	
XIII	13β(H)-Azorellane	C_20_H_34_	274	I		26
XIV	13α(H)-Azorellane	C_20_H_34_	274	I		15
XV	13β(H)-Mulinane	C_20_H_36_	276	I		11
XVI	13α(H)-Mulinane	C_20_H_36_	276	I		8
XVII	13-Hydroxyazorellanes	C_20_H_34_O	290	L		14
XVIII	13α-Hydroxymulinane	C_20_H_36_O	292	L		22
XIX	13β(H)-Mulina-11,14-dien-20-oic acid	C_20_H_30_O_2_	302	S	10	
XX	Mulina-11,13-dien-20-oic acid	C_20_H_30_O_2_	302	S	100	
XXI	Azorell-13-en-20-oic acid	C_20_H_30_O_2_	302	S	17	
XXII	13β(H)-Azorellan-20-oic acid	C_20_H_32_O_2_	304	S		5
XXIII	13α(H)-Azorellan-20-oic acid	C_20_H_32_O_2_	304	S		30
XXIV	13β(H)-Mulinan-20-oic acid	C_20_H_34_O_2_	306	S		44
XXV	13α(H)-Mulinan-20-oic acid	C_20_H_34_O_2_	306	S		100
XXVI	Mulinol	C_20_H_34_O_2_	306	L	7	
XXVII	Dihydromulinol	C_20_H_36_O_2_	308	I		6
XXVIII	13α-Hydroxymulin-11-en-20-oic acid	C_20_H_32_O_3_	320	S	8	
XXIX	13α-Hydroxymulinan-20-oic acid	C_20_H_34_O_3_	322	S		25
XXX	20-Acetoxymulina-11,13-diene	C_22_H_34_O_2_	330	L	15	
XXXI	13α,14β-Dihydroxymulin-11-en-20-oic acid	C_20_H_30_O_4_	336	L	11	
XXXII	13α,14β-Dihydroxymulinan-20-oic acid	C_20_H_34_O_4_	338	I		32
XXXIII	Azorellanol	C_22_H_36_O_3_	348	L	16	12

^a^ Structures are shown in Appendix A. ^b^ Listed as the natural products, polar compounds analyzed as methyl esters and/or trimethylsilyl derivatives. ^c^ Identification: I = interpretation and correlation of MS fragmentation pattern and GC retention time as derivative from standard, T = suggested interpretation based on MS and GC retention time only, S = standard, L = literature citation, n = number of analyses.

**Figure A1:**
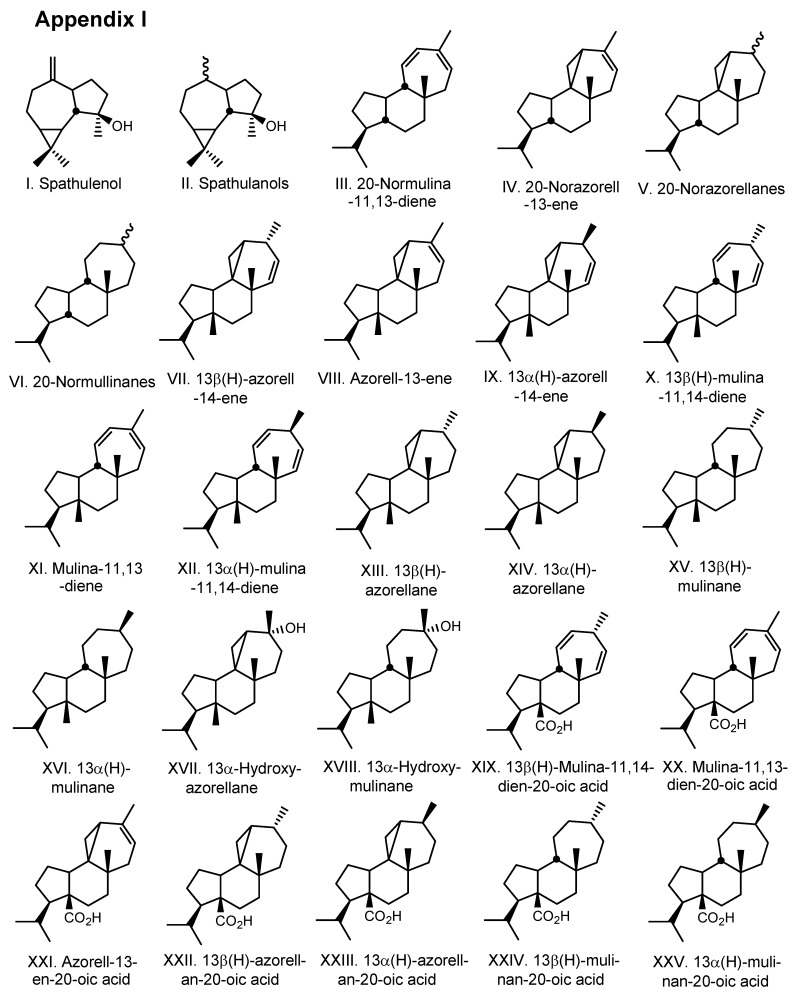

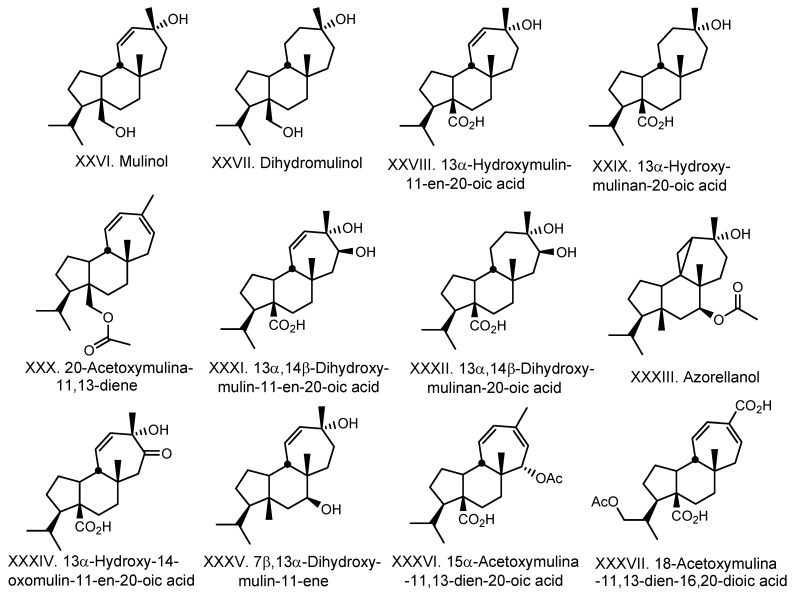
Chemical structures cited and listed in Table 1.

## Supplementary Materials

Additional mass spectra of related and derivatized natural products are collected in the Supplemental Materials section available with this article.
