# Supplementary material for: Mulinane and Azorellane Diterpenoid Biomarkers by GC-MS from a Representative Apiaceae (Umbelliferae) Species of the Andes
Source: Molecules. 2019 Feb 14;24(4):684. doi: 10.3390/molecules24040684 (PMC6412730; doi:10.3390/molecules24040684)
Supplement: Supplementary file 1 [file molecules-24-00684-s001.pdf]

Supplemental Materials for

Mulinane and Azorellane Diterpenoid Biomarkers by GC-MS from a Representative Apiaceae (Umbelliferae) Species of the Andes

Bernd R.T. Simoneit [1,*], Daniel R. Oros [2], Rudolf Jaffé [3], Alexandra Didyk-Peña [4,*], Carlos Areche [5], Beatriz Sepúlveda [6] and Borys M. Didyk [7]

1 Department of Chemistry, College of Science, Oregon State University, Corvallis, OR 97331, USA
2 Consultant, 72 Marina Lakes Drive, Richmond, CA 94804, USA; daniel.r.oros@gmail.com
3 Southeast Environmental Research Center and Department of Chemistry and Biochemistry, Florida International University, 3000 NE 151st Street, North Miami, FL 33181, USA; jaffer@fiu.edu
4 Escuela de Química y Farmacia, Facultad de Medicina, Universidad Andres Bello, Viña del Mar 2520000, Chile
5 Departamento de Química, Facultad de Ciencias, Universidad de Chile, Casilla 653, Santiago 8320000, Chile; areche@uchile.cl
6 Departamento de Ciencias Químicas, Universidad Andres Bello, Viña del Mar 2520000, Chile; bsepulveda@uc.cl
7 Consultant, Casilla 942, Viña del Mar 2520000, Chile; bmdidyk@gmail.com
* Correspondence: Correspondence: simonebe@oregonstate.edu (B.R.T.S.); maria.didyk@unab.cl (A.D.-P.); Tel.: +1-541-737-2081 (B.R.T.S.)

Contents:

Standards

Figure SM-1. Mass spectra of derivatives, related standards and unknown compounds in *A. compacta* resins.

Figure SM-2. Fragmentation schemes indicating the key ions for the mass spectra of the diterpenoids in *A. compacta* resins.

Standards

The mass spectra of primary, secondary (hydrogenated) and surrogate standards are collected in Figure SM-1. The following standard compounds were not detected in the resin sample of this report, but are provided for future reference: 13α-hydroxy-14-oxomulin-11-en-20-oic acid (Fig. SM-1j,w,z), 15α-acetoxymulina-11,13-dien-20-oic acid (Fig. SM-1x), 7β,13α-dihydroxymulin-11-ene (Fig. SM-1s), and 18-acetoxymulina-11,13-dien-16,20-dioic acid (Fig. SM-1aa).

Figure SM-1. Mass spectra of derivatives, related standards and unknown compounds in *A. compacta* resins. The KI values relative to *n*-alkanes on a DB-5 column are given in parentheses on the mass spectra:

(a) α-spathulanol (II),
(b) β-spathulanol (II),
(c) spathulanol-TMS (II),
(d) mulinolic acid (XXVIII, standard),
(e) 13α-hydroxyazorellan-20-oic acid (standard, with XXVII),
(f) 7β-acetoxyazorell-13-ene (interpreted based on XXXIII),
(g) 7α-acetoxyazorell-13-ene (interpreted based on XXXIII),
(h) methyl mulinolate (XXVIII, standard),

(i) methyl 13α-methoxymulin-11-en-20-oate (XXVIII, standard),
(j) methyl 13α-hydroxy-14-oxomulin-11-en-20-oate (XXXIV, standard),
(k) 13-*epi*-azorellanol (XXXIII),
(l) 7β-hydroxyazorellane-TMS,
(m) 13α-hydroxyazorellane-TMS (XVII),
(n) 13α-hydroxymulinane-TMS (XVIII),
(o) 13β(H)-mulina-11,14-dien-20-oic acid-TMS (XIX, standard),
(p) mulina-11,13-dien-20-oic acid-TMS (XX, standard),
(q) azorell-13-en-20-oic acid-TMS (XXI, standard),
(r) 13β(H)-azorellan-20-oic acid-TMS (XXII),
(s) 13α(H)-azorellan-20-oic acid-TMS (XXIII),
(t) 13β(H)-mulinan-20-oic acid-TMS (XXIV, standard),
(u) 13α(H)-mulinan-20-oic acid-TMS (XXV, standard),
(v) azorellanol-TMS (XXXIII, standard),
(w) 13α-hydroxy-14-oxomulin-11-en-20-oic acid-20TMS (XXXIV, standard),
(x) 15α-acetoxymulina-11,13-dien-20-oic acid-TMS (XXXVI, standard),
(y) 7β,13α-dihydroxymulin-11-ene-diTMS (XXXV, standard),
(z) 13α-hydroxy-14-oxomulin-11-en-20-oic acid-diTMS (XXXIV, standard),
(aa) 18-acetoxymulina-11,13-dien-16,20-dioic acid-diTMS (XXXVII, standard),
(bb) unknown $C_{20}H_{34}$,
(cc) unknown $C_{20}H_{32}$,
(dd) unknown $C_{20}H_{32}$, and
(ee) unknown $C_{20}H_{32}$.

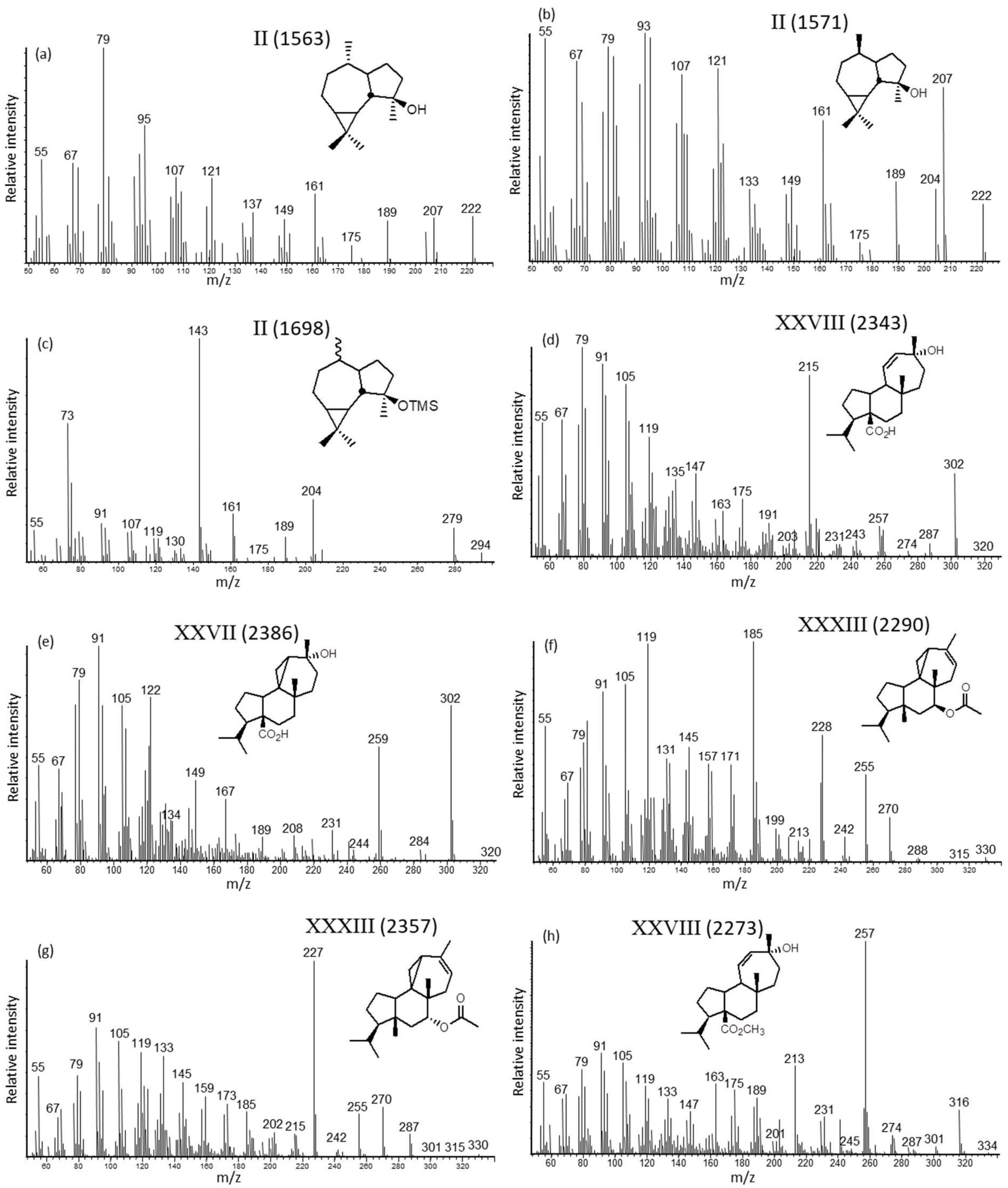
(a)
II (1563)
Relative intensity
m/z
OH
55 67 79 95 107 121 137 149 161 175 189 207 222
(b)
II (1571)
Relative intensity
m/z
OH
55 67 79 93 107 121 133 149 161 175 189 204 207 222
(c)
II (1698)
Relative intensity
m/z
OTMS
55 73 91 107 119 130 143 161 175 189 204 279 294
(d)
XXVIII (2343)
Relative intensity
m/z
OH
CO_2H
55 67 79 91 105 119 135 147 163 175 191 203 215 231 243 257 274 287 302 320
(e)
XXVII (2386)
Relative intensity
m/z
OH
CO_2H
55 67 79 91 105 122 134 149 167 189 208 231 244 259 284 302 320
(f)
XXXIII (2290)
Relative intensity
m/z
55 67 79 91 105 119 131 145 157 171 185 199 213 228 242 255 270 288 315 330
(g)
XXXIII (2357)
Relative intensity
m/z
55 67 79 91 105 119 133 145 159 173 185 202 215 227 242 255 270 287 301 315 330
(h)
XXVIII (2273)
Relative intensity
m/z
OH
CO_2CH_3
55 67 79 91 105 119 133 147 163 175 189 201 213 231 245 257 274 287 301 316 334

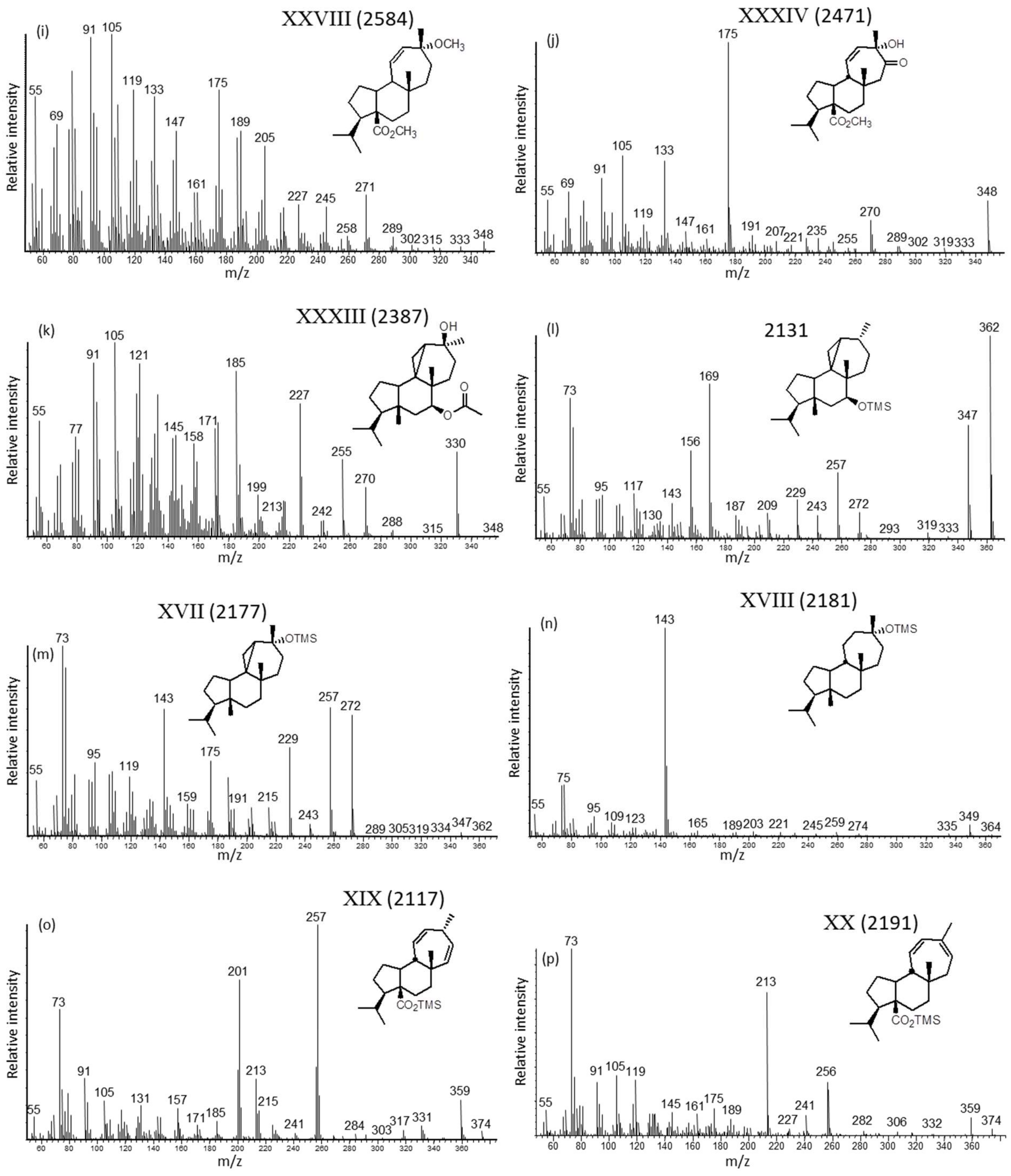
XXVIII (2584)
(i)
55 69 91 105 119 133 147 161 175 189 205 227 245 258 271 289 302 315 333 348
OCH3
CO2CH3
XXXIV (2471)
(j)
175
55 69 91 105 119 133 147 161 191 207 221 235 255 270 289 302 319 333 348
OH
O
CO2CH3
XXXIII (2387)
(k)
55 77 91 105 121 145 158 171 185 199 213 227 242 255 270 288 315 330 348
OH
2131
(l)
55 73 95 117 130 143 156 169 187 209 229 243 257 272 293 319 333 347 362
OTMS
XVII (2177)
(m)
55 73 95 119 143 159 175 191 215 229 243 257 272 289 305 319 334 347 362
OTMS
XVIII (2181)
(n)
55 75 95 109 123 143 165 189 203 221 245 259 274 335 349 364
OTMS
XIX (2117)
(o)
55 73 91 105 131 157 171 185 201 213 215 241 257 284 303 317 331 359 374
CO2TMS
XX (2191)
(p)
55 73 91 105 119 145 161 175 189 213 227 241 256 282 306 332 359 374
CO2TMS
Relative intensity
m/z

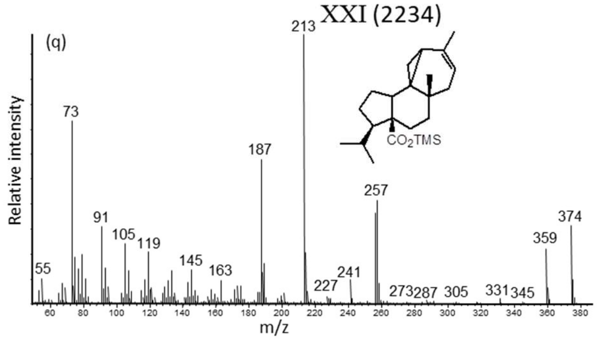
XXI (2234)
(q)
Relative intensity
m/z
CO2TMS
55 73 91 105 119 145 163 187 213 227 241 257 273 287 305 331 345 359 374

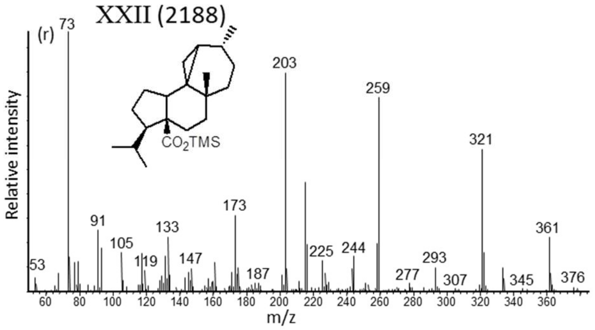
XXII (2188)
(r)
Relative intensity
m/z
CO2TMS
53 73 91 105 119 133 147 173 187 203 225 244 259 277 293 307 321 345 361 376

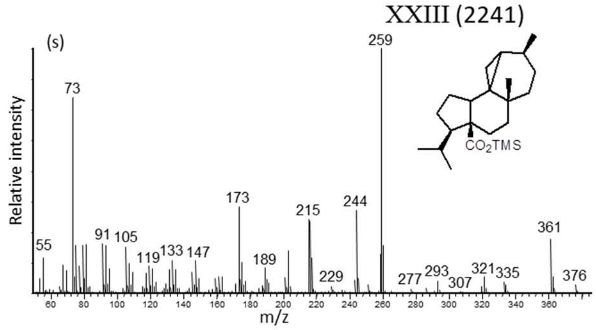
XXIII (2241)
(s)
Relative intensity
m/z
CO2TMS
55 73 91 105 119 133 147 173 189 215 229 244 259 277 293 307 321 335 361 376

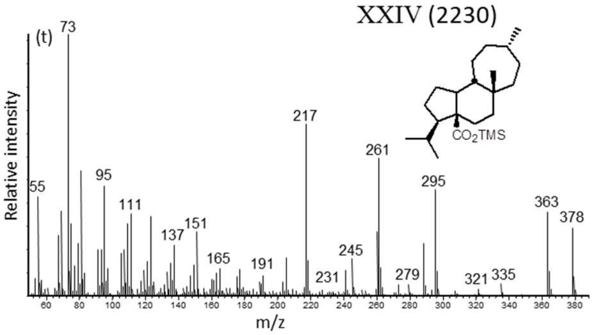
XXIV (2230)
(t)
Relative intensity
m/z
CO2TMS
55 73 95 111 137 151 165 191 217 231 245 261 279 295 321 335 363 378

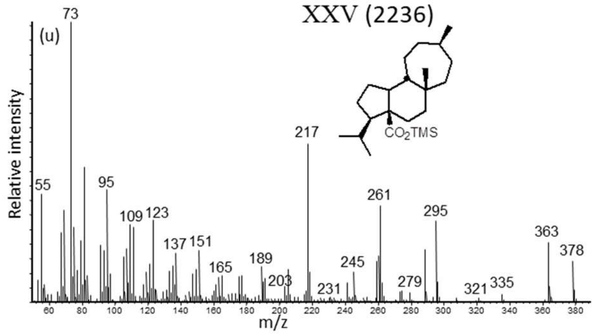
XXV (2236)
(u)
Relative intensity
m/z
CO2TMS
55 73 95 109 123 137 151 165 189 203 217 231 245 261 279 295 321 335 363 378

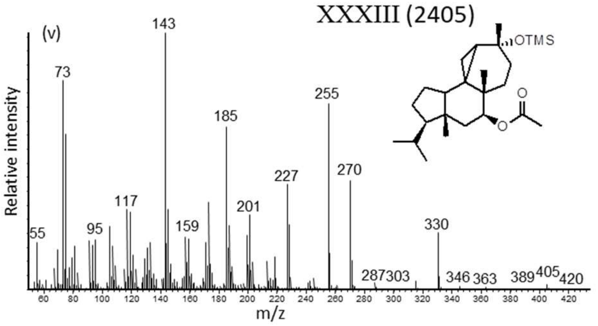
XXXIII (2405)
(v)
Relative intensity
m/z
OTMS
55 73 95 117 143 159 185 201 227 255 270 287 303 330 346 363 389 405 420

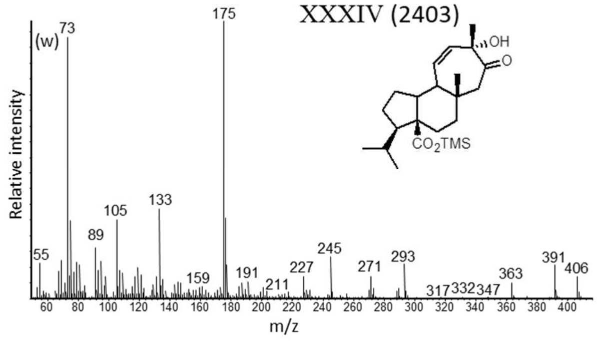
XXXIV (2403)
(w)
Relative intensity
m/z
OH
O
CO2TMS
55 73 89 105 133 159 175 191 211 227 245 271 293 317 332 347 363 391 406

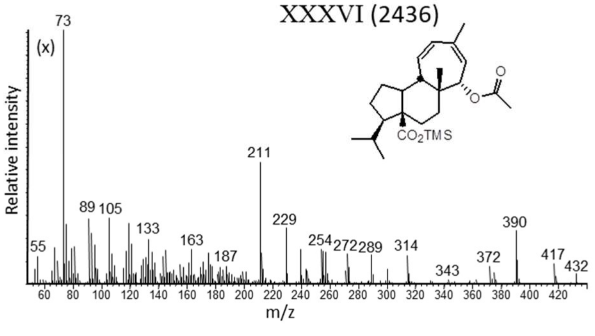
XXXVI (2436)
(x)
Relative intensity
m/z
O
CO2TMS
55 73 89 105 133 163 187 211 229 254 272 289 314 343 372 390 417 432

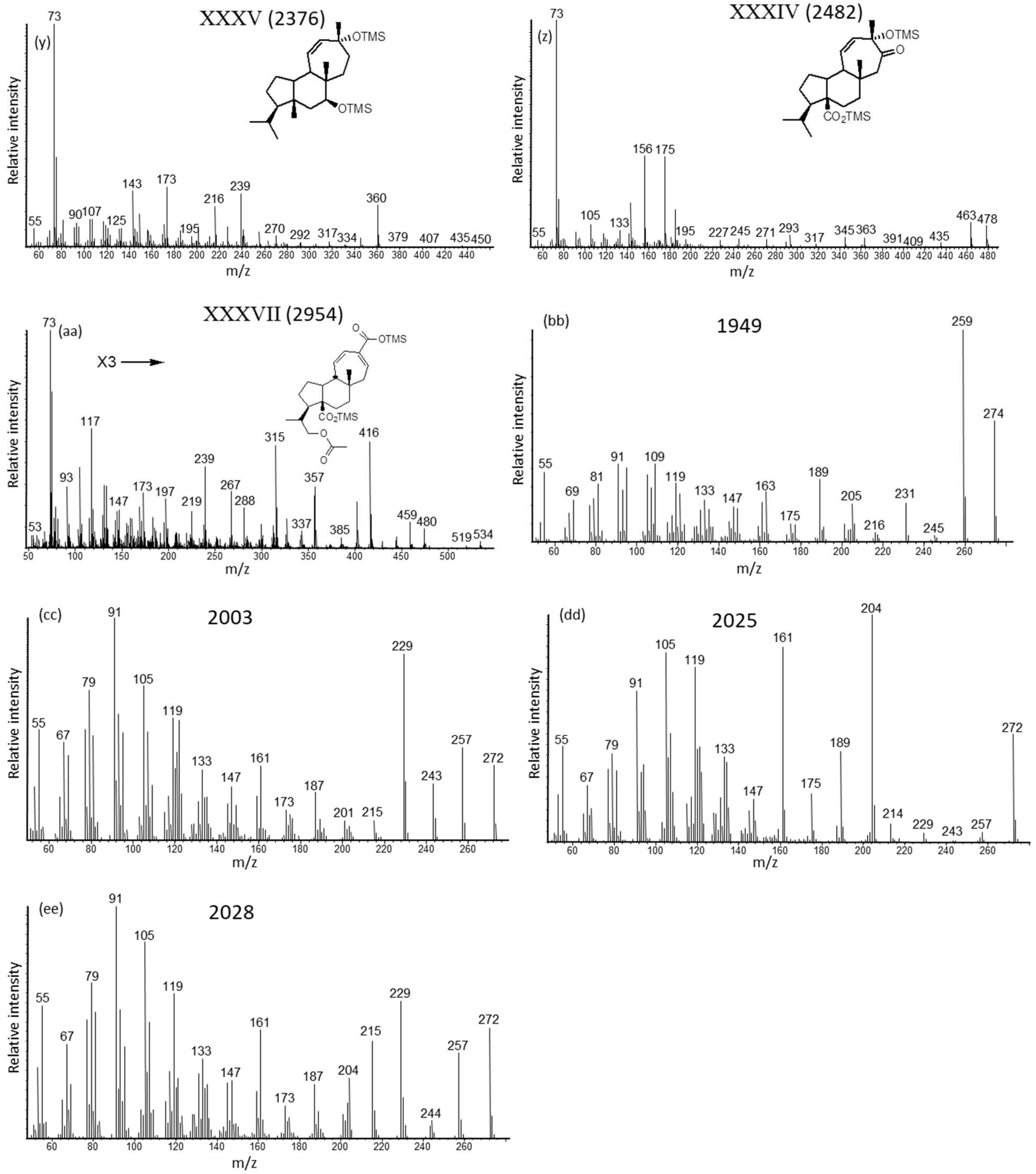
XXXV (2376)
(y)
OTMS
OTMS
Relative intensity
m/z
73 55 90 107 125 143 173 195 216 239 270 292 317 334 360 379 407 435 450
XXXIV (2482)
(z)
OTMS
O
CO2TMS
73 55 105 133 156 175 195 227 245 271 293 317 345 363 391 409 435 463 478
XXXVII (2954)
(aa)
X3
OTMS
CO2TMS
73 53 93 117 147 173 197 219 239 267 288 315 337 357 385 416 459 480 519 534
(bb)
1949
55 69 81 91 109 119 133 147 163 175 189 205 216 231 245 259 274
(cc)
2003
55 67 79 91 105 119 133 147 161 173 187 201 215 229 243 257 272
(dd)
2025
55 67 79 91 105 119 133 147 161 175 189 204 214 229 243 257 272
(ee)
2028
55 67 79 91 105 119 133 147 161 173 187 204 215 229 244 257 272

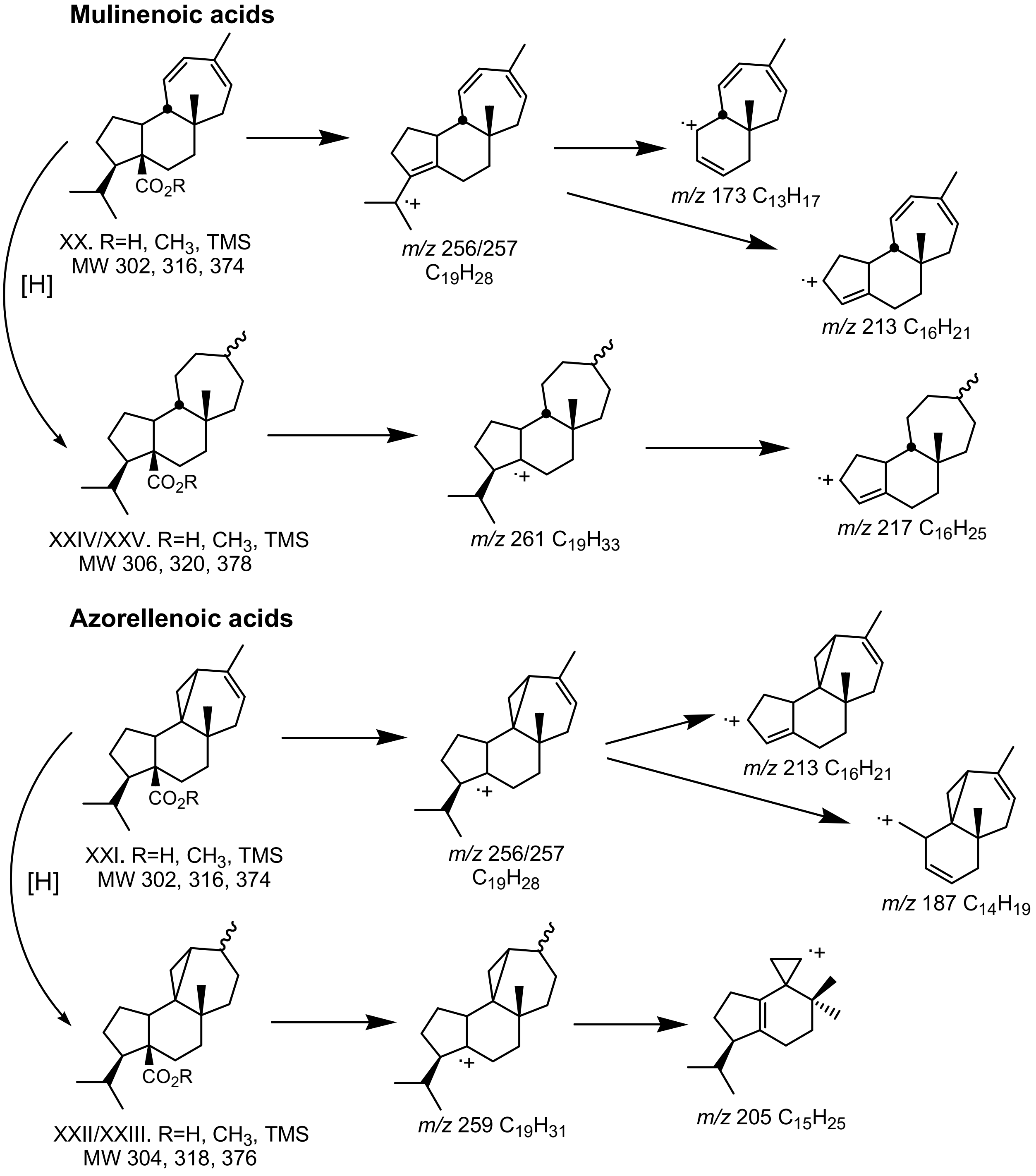

Figure SM-2a. Fragmentation scheme indicating the key ions for the mass spectra of the diterpenoid acids in *A. compacta* resin (cf. Fig. 4 in text).

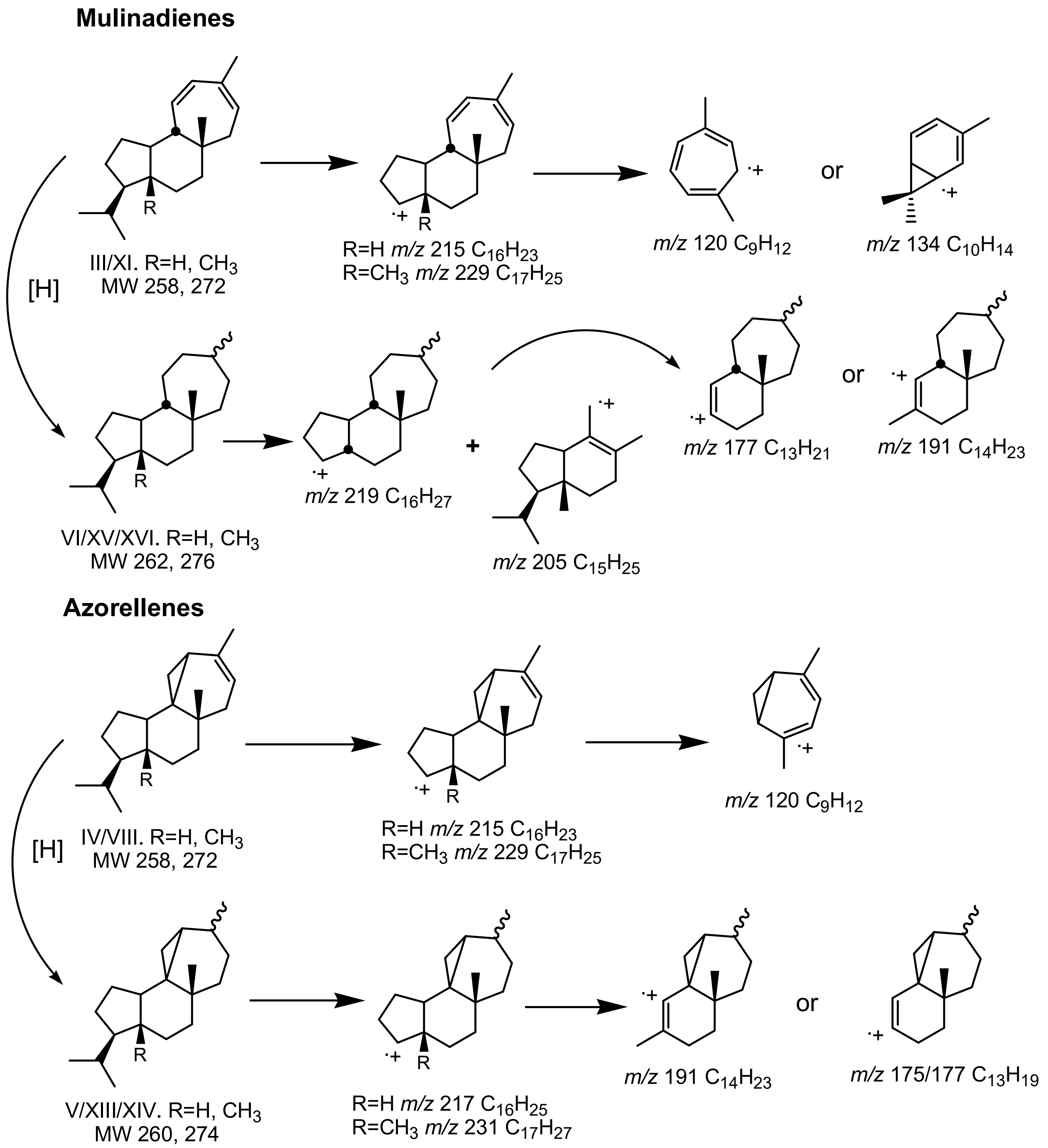

Figure SM-2b. Fragmentation scheme indicating the key ions for the mass spectra of the diterpenoid hydrocarbons in *A. compacta* resin (cf. Fig. 3 in text).
